# Changing Epidemiology, Healthcare-Associated Infections, and Outcomes in Infective Endocarditis: A Five-Year Retrospective Study from a Tertiary Cardiovascular Center

**DOI:** 10.3390/medicina62061028

**Published:** 2026-05-26

**Authors:** Adelina Matei, Grigore Tinică, Alberto Bacușcă, Mihail Enache, Andrei Țăruș, Mihaela Cătălina Luca, Gabriela Jugănariu, Doina Azoicăi

**Affiliations:** 1 Faculty of Medicine, “Grigore T. Popa” University of Medicine and Pharmacy, 700115 Iasi, Romania; grigore.tinica@umfiasi.ro (G.T.); alberto-bacusca@email.umfiasi.ro (A.B.); enache.mihail@umfiasi.ro (M.E.); catalina_luca2006@yahoo.com (M.C.L.); doina.azoicai@gmail.com (D.A.); 2 “George I.M. Georgescu” Institute for Cardiovascular Diseases, 700503 Iasi, Romania; 3 “Saint Parascheva” Clinical Hospital of Infectious Diseases, 700116 Iasi, Romania; gabriela_juganariu@yahoo.com

**Keywords:** infective endocarditis, HAIs, mortality, acute kidney injury, sepsis, cohort

## Abstract

*Background and Objectives*: Infective endocarditis (IE) remains a major clinical challenge. It carries high morbidity and mortality, despite advances in diagnostic and therapeutic methods. This study aimed to evaluate the epidemiological profile, microbiological characteristics, complications, and predictors of adverse outcomes among patients with IE treated at a tertiary cardiovascular center in Romania over 5 years. *Materials and Methods*: We conducted a retrospective study including 156 patients diagnosed with IE between January 2020 and December 2024. We analyzed demographic data, comorbidities, microbiological findings, treatment strategies, complications, and in-hospital outcomes. *Results:* The cohort was predominantly male (76.3%), with a mean age of 58.5 years. Native valve endocarditis was the most frequent form (80.1%). Streptococci were the most commonly identified pathogens, followed by enterococci and staphylococci. Complications occurred in 74.4% of patients. Heart failure (70.5%), acute kidney injury (37.2%), and embolic events (32.7%) were most frequent. Healthcare-associated infective endocarditis (HAIE) was seen in 10.3% of patients. Additional healthcare-associated infections (HAIs) occurred in 26.9% of patients and were associated with longer hospital stays (21.7 vs. 13.5 days; *p* < 0.001). Use of a central venous catheter independently predicted HAI development (adjusted OR, 3.89; 95% CI, 1.08–14.06; *p* = 0.038). The in-hospital mortality rate was 16.7%. Acute kidney injury and sepsis were the strongest factors associated with in-hospital mortality. *Conclusions*: IE remains associated with a high burden of complications and in-hospital mortality. HAIs complicate the clinical course and are closely linked to invasive device use. Mortality is mainly driven by systemic disease severity, especially acute kidney injury and sepsis. These findings highlight the importance of infection prevention, prompt risk stratification, and coordinated multidisciplinary care to improve outcomes in patients with IE.

## 1. Introduction

IE remains a severe and potentially life-threatening condition with persistently high morbidity and mortality, characterized by infection of the endocardial surface, most commonly involving the cardiac valves. Although relatively uncommon, its incidence has gradually increased over recent decades, currently estimated at approximately 3–10 cases per 100,000 population annually [1,2]. This trend likely reflects both improved diagnostic capabilities and changing patient demographics.

Over time, the epidemiology of IE has undergone a notable transformation. The classical profile of younger patients with rheumatic heart disease has progressively shifted toward older individuals with multiple comorbidities, including degenerative valvular disease, diabetes mellitus, and chronic kidney disease [1,3]. In parallel, the widespread use of invasive medical procedures, prosthetic valves, and intracardiac devices has led to an increasing proportion of healthcare-associated endocarditis cases [4,5].

A meaningful shift in microbiological etiology has also been observed in recent decades. While viridans group streptococci were historically the predominant causative agents, staphylococci—particularly *Staphylococcus aureus*—and enterococci have become the leading pathogens in contemporary cohorts [6,7,8]. This change is clinically relevant, as these microorganisms are associated with more aggressive disease, higher complication rates, and worse outcomes.

Despite advances in diagnostic techniques, including echocardiography and modern imaging modalities, IE remains a diagnostic challenge due to its heterogeneous and often nonspecific clinical presentation [9]. The modified Duke criteria remain the foundation of diagnosis; however, their sensitivity may be limited in certain clinical scenarios, necessitating integration with high-resolution imaging and clinical judgment [10].

IE continues to be associated with substantial morbidity and mortality, with in-hospital mortality rates regularly ranging between 15% and 30% [2,11]. Prognosis is determined by multiple factors, including patient characteristics, comorbidities, causative microorganisms, and complications such as heart failure, embolic events, or sepsis [11,12]. Early identification of high-risk patients is therefore key to optimizing clinical strategies. Given the complexity of this condition, current management underlines an interdisciplinary approach involving cardiologists, infectious disease specialists, microbiologists, and cardiac surgeons. The implementation of dedicated “endocarditis teams” has been associated with enhanced outcomes and more appropriate therapeutic decision-making [13].

In the context of evolving epidemiology and persistent high mortality, continuous regional evaluation of IE remains crucial. The present study aims to assess the epidemiological, microbiological, and clinical characteristics of infective endocarditis over 5 years at a tertiary cardiovascular center, with a focus on recognizing trends and factors associated with patient clinical outcomes.

## 2. Materials and Methods

### 2.1. Study Design and Population

We conducted a retrospective, observational, single-center study of patients diagnosed with infective endocarditis (IE) admitted to a tertiary cardiovascular center in Romania between January 2020 and December 2024. All patients meeting the diagnostic criteria for IE during this period were eligible, and 156 were included in the final analysis.

### 2.2. Diagnostic Criteria

The diagnosis of infective endocarditis was based on the modified Duke criteria. Clinical, microbiological, and echocardiographic findings were integrated. Cases were classified as definite or possible IE based on these criteria.

### 2.3. Data Collection

Clinical data were collected retrospectively from hospital records and electronic databases. These incorporated demographic characteristics, admission presentation, comorbidities, cardiovascular risk factors, microbiological findings, echocardiographic features, treatment strategies, and in-hospital outcomes.

### 2.4. Definitions

Healthcare-associated infective endocarditis (HAIE) was defined as IE in patients with recent or ongoing healthcare exposure before diagnosis. Healthcare exposure included recent hospitalization, frequent outpatient or home-based care, hemodialysis, residence in a long-term care facility, or exposure to invasive procedures, according to the ESC 2023 and standard definitions of healthcare-associated infections.

Persistent bacteremia was defined as persistently positive follow-up blood cultures despite 48–72 h of appropriate antimicrobial therapy. Severe clinical presentation was characterized by septic shock, cardiogenic shock, coma, or multiorgan failure at the time of admission.

Healthcare-associated infections (HAIs) and HAIEs were analyzed as distinct entities. HAIE specifically referred to IE with prior healthcare exposure, whereas HAIs described other healthcare-associated infections. Examples of HAIs included pneumonia, bloodstream infections, surgical site infections, or catheter-related infections that were not persistent, relapsed, or recurrent episodes of the primary IE. Secondary HAIs were defined as infections separate from the primary IE episode, excluding persistent infection, relapse, or recurrence of IE when these were microbiologically or clinically attributable to the initial infective episode.

Cardiac predisposing factors were defined as pre-existing structural or functional abnormalities that increase risk for infective endocarditis. These included degenerative valvular disease, prior IE, mitral valve prolapse, bicuspid aortic valve, hypertrophic cardiomyopathy, prosthetic valves, or intracardiac devices.

### 2.5. Outcomes

The primary outcome of the study was in-hospital mortality, which served as the central focus for subsequent statistical analyses.

Secondary outcomes included:

• occurrence of major complications;
• need for surgical intervention;
• length of hospital stay.

### 2.6. Statistical Analysis

Statistical analyses were performed using XLSTAT version 2019.4 (Addinsoft, Paris, France) for Microsoft Excel (Microsoft Corporation, Redmond, WA, USA). Continuous variables were expressed as mean ± standard deviation or median (interquartile range). Categorical variables were presented as frequencies and percentages. To compare groups, Student’s *t*-test or Mann–Whitney U test was used for continuous variables. For categorical variables, the chi-square test or Fisher’s exact test was applied.

Univariate analyses identified factors associated with in-hospital mortality and HAIs. Odds ratios (ORs) with 95% confidence intervals (CIs) were calculated. A two-sided *p*-value < 0.05 was considered statistically significant.

Variables considered clinically relevant a priori and/or statistically significant in univariate analysis were included in multivariable logistic regression models to reduce model overfitting.

### 2.7. Ethical Considerations

The study was conducted in accordance with the principles of the Declaration of Helsinki. Given the study’s retrospective nature, informed consent was waived. Patient data were anonymized before analysis to ensure confidentiality.

## 3. Results

A total of 156 patients diagnosed with IE per the modified Duke criteria were included in this retrospective study. The cohort was predominantly male, with 119 men (76.3%) and 37 women (23.7%). The mean age was 58.5 years. Women were older than men (60.9 vs. 57.8 years). Most patients were in the 60–69-year age group (*n* = 49), followed by those aged 70 years or older (*n* = 36) and those aged 50–59 years (*n* = 35). Most patients were from urban areas (*n* = 88, 56.4%).

A total of 45.5% of patients were referred from other healthcare facilities, reflecting the center’s tertiary referral function and the complexity of the cases managed. The mean hospitalization duration at the tertiary center was 17.62 days, underscoring the significant healthcare burden associated with infective endocarditis.

### 3.1. Annual Distribution of Cases

The annual number of infective endocarditis cases fluctuated during the study period, ranging from 15 cases (9.6%) in 2020 to a peak of 40 cases (25.6%) in 2022. Specifically, there were 15 cases (9.6%) in 2020, 32 (20.5%) in 2021, 40 (25.6%) in 2022, 32 (20.5%) in 2023, and 37 (23.7%) in 2024. The lower case count in 2020 coincided with the onset of the COVID-19 pandemic, while higher annual case numbers were observed in subsequent years.

### 3.2. Type of Endocarditis and Acquisition Setting

Based on the underlying cardiac structure involved, infective endocarditis was classified as native valve endocarditis (NVE), prosthetic valve endocarditis (PVE), or device-related infective endocarditis, in accordance with the 2023 European Society of Cardiology Guidelines. NVE, defined as an infection affecting native cardiac valves, was the predominant form, identified in 125 patients (80.1%). PVE, defined as infective endocarditis involving mechanical or biological prosthetic valves, was diagnosed in 24 patients (15.4%). Cardiac device-related infective endocarditis, defined as infection associated with cardiac implantable electronic devices, was identified in 7 patients (4.5%).

Based on the mode of acquisition, healthcare-associated infective endocarditis (HAIE) was seen in 16 patients (10.3%). Most cases were community-acquired (*n* = 136, 87.2%). In 4 patients (2.6%), the acquisition mode was undetermined.

A presumed portal of entry was identified in 95 patients (60.9%). The most common entry sites were cutaneous, oral or dental, and gastrointestinal.

### 3.3. Comorbidities, Cardiac Predisposing Factors, and Risk Exposures

Comorbidities affected 95.5% of patients, with cardiovascular disease being most common. Other frequent comorbidities included chronic kidney disease, diabetes mellitus, gastrointestinal and pulmonary disorders. Cardiac predisposing factors were present in 73% of patients, the most common being degenerative valvular disease (17.9%), followed by previous infective endocarditis (12.8%), mitral valve prolapse (11.5%), bicuspid aortic valve (11.5%), and hypertrophic cardiomyopathy (10.9%).

Additional risk exposures included prosthetic heart valves, intracardiac devices, and recent invasive procedures, although cardiac catheterization was relatively uncommon. Intravenous drug use was identified in only 1 patient (0.6%).

In summary, the cohort exhibited a high prevalence of comorbidities, frequent structural cardiac abnormalities, and significant healthcare-related exposures, as detailed in Table 1.

**Table 1:** Baseline characteristics of the study population.

Variable	Value
Age, mean (years)	58.5
Male sex, *n* (%)	119 (76.3)
At least one comorbidity, *n* (%)	149 (95.5)
Cardiovascular disease, *n* (%)	144 (92.3)
Chronic kidney disease, *n* (%)	54 (34.6)
Diabetes mellitus, *n* (%)	38 (24.4)
Cardiac predisposing factor *, *n* (%)	114 (73.0)

* Cardiac predisposing factors included pre-existing structural or functional cardiac abnormalities associated with increased susceptibility to infective endocarditis, such as degenerative valvular disease, prior infective endocarditis, mitral valve prolapse, bicuspid aortic valve, hypertrophic cardiomyopathy, prosthetic valves, or intracardiac devices.

### 3.4. Clinical Presentation

The clinical presentation of infective endocarditis was heterogeneous, including both acute and subacute forms at admission. Most patients (118 cases, 75.6%) exhibited a subacute onset, whereas 38 patients (24.4%) presented with an acute form.

Fever occurred in 85 patients (54.5%), and chills were reported in 35 patients (22.4%). Constitutional symptoms were prevalent, including fatigue in 113 patients (72.4%), dyspnea in 101 (64.7%), anorexia in 28 (17.9%), profuse sweating in 28 (17.9%), and weight loss in 16 (10.3%). Cardiac involvement was frequently documented, with a heart murmur present in 111 patients (71.2%). Peripheral and immunological manifestations were less common. Cutaneous phenomena were observed in 19 patients (12.2%), vascular manifestations in 38 patients (24.4%), and glomerulonephritis in 38 patients (24.4%). Neurological symptoms, such as headache, were reported in 18 patients (11.5%).

Overall, the clinical presentation was frequently nonspecific, with constitutional and cardiac symptoms predominating. These findings highlight the complex and variable nature of infective endocarditis at initial presentation.

### 3.5. Echocardiographic Findings

Vegetations were identified in 143 patients (91.7%), confirming the high diagnostic yield of echocardiography in this cohort.

The aortic valve was the most frequently involved site (*n* = 92), with 73 cases affecting native valves and 19 involving prosthetic valves. The mitral valve was involved in 35 cases, including 2 prosthetic valves. Right-sided involvement was less common, with the tricuspid valve affected in 4 cases and the pulmonary valve in 1 case. Combined aortic and mitral involvement occurred in 17 patients. Device-related vegetations on intracardiac leads were identified in 7 cases.

Most vegetations were mobile, observed in 78.2% of cases. Regarding size, 60.8% of the vegetations measured less than 10 mm, while 26.9% were classified as large (greater than 10 mm). Multiple vegetations were present in 19 patients (12.1%).

**Figure 1:**
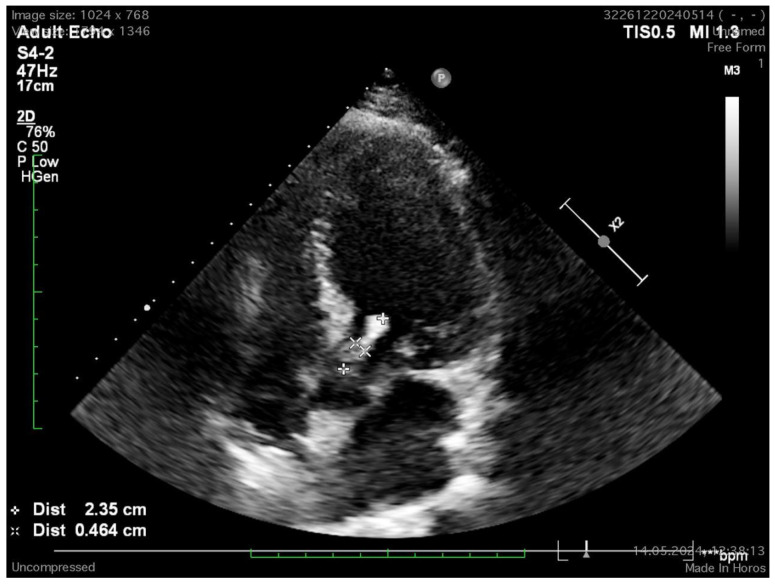
Representative echocardiographic image demonstrating valvular vegetation in infective endocarditis.

Representative echocardiographic images illustrating typical valvular vegetations and lesion morphology are presented in Figure 1.

### 3.6. Laboratory Findings

Laboratory abnormalities at admission were highly prevalent, reflecting the significant systemic inflammatory and infectious burden associated with infective endocarditis (Table 2).

Anemia and leukocytosis were the most frequent abnormalities, each affecting more than three-quarters of patients. Elevated C-reactive protein levels were also observed in the majority of cases, further supporting the cohort’s pronounced inflammatory profile. Platelet abnormalities were less common but remained clinically relevant, with both thrombocytopenia and thrombocytosis identified in a subset of patients.

These findings demonstrate the substantial hematological and inflammatory disturbances characteristic of infective endocarditis. Subsequent analyses revealed that leukocytosis was associated with increased in-hospital mortality in univariate analysis.

### 3.7. Etiological Agents of Infective Endocarditis

An etiological agent was identified in 105 patients (67.3%), including 103 with positive blood cultures and 2 with *Coxiella burnetii* detected by serological testing (Table 3). Fifty-one patients (32.7%) had culture-negative infective endocarditis, which highlights the diagnostic challenges of microbiologically unconfirmed cases. Among confirmed cases, streptococci were the most common pathogens, followed by enterococci and staphylococci. Gram-negative pathogens and *Coxiella burnetii* were rare.

Species-level analysis further delineated the distribution of pathogens within the principal microbiological groups. Among streptococci, *Streptococcus gallolyticus* was most frequently identified (*n* = 16), followed by viridans group streptococci, including *Streptococcus viridans* (*n* = 8), *Streptococcus mitis* (*n* = 6), and *Streptococcus oralis* (*n* = 2), as well as less common species such as *Streptococcus anginosus* and *Streptococcus sanguinis*.

Within the enterococcal group, *Enterococcus faecalis* was the predominant species (*n* = 9), followed by *Enterococcus faecium* (*n* = 4). Fifteen isolates were reported as *Enterococcus* spp., without further species-level identification. Among staphylococci, *Staphylococcus aureus* was a major pathogen, with both methicillin-resistant (MRSA, *n* = 9) and methicillin-sensitive (MSSA, *n* = 5) isolates identified. *Staphylococcus epidermidis* (*n* = 6) and *Staphylococcus hominis* (*n* = 4) were also detected, as well as other MR-CoNS (*n* = 3), which were more frequently associated with prosthetic valve and device-related infections.

A substantial proportion of patients were referred from other healthcare facilities and had received prior antibiotic therapy, which may partially account for the relatively high proportion of culture-negative infective endocarditis observed in this cohort. Streptococci, enterococci, and staphylococci, consistent with contemporary epidemiological patterns of infective endocarditis, dominated the microbiological distribution.

### 3.8. Antimicrobial Therapy

Antimicrobial therapy began immediately after blood cultures were collected and was subsequently adjusted based on microbiological findings, susceptibility testing, infectious foci, and established guidelines. When a specific pathogen was identified, treatment was guided by its susceptibility profile. Concomitant infections required additional modifications based on microbiological and clinical data.

A considerable proportion of patients received antibiotic therapy before admission, frequently as empirical regimens initiated at referring healthcare facilities. Such pre-treatment likely contributed to the heterogeneity of antimicrobial strategies observed within the cohort and to the relatively high incidence of culture-negative infective endocarditis. Accordingly, Table 4 summarizes the antimicrobial regimens most frequently administered within the overall cohort.

Among patients with culture-negative infective endocarditis, empirical therapy most commonly consisted of broad-spectrum combinations, particularly ceftriaxone plus vancomycin (Table 5). The duration of antimicrobial therapy typically ranged from 4 to 6 weeks of intravenous administration, determined by the causative organism, valve type, and presence of complications. Extended treatment courses were frequently necessary for prosthetic valve endocarditis and persistent infections.

In addition to therapy duration, distinct pathogen-specific therapeutic patterns were identified. Streptococcal infections were primarily managed with beta-lactam–based regimens, enterococcal infections with ampicillin-based combinations, and staphylococcal infections with glycopeptide-based therapy, particularly when methicillin resistance was suspected or confirmed. Prosthetic valve infective endocarditis often requires broader combination regimens, usually incorporating rifampicin-based strategies. The diversity of antimicrobial regimens underscores the microbiological heterogeneity of infective endocarditis and the complexity of individualized therapeutic management.

### 3.9. Surgical Treatment

Surgical management was required in patients with advanced valvular involvement and severe disease-related complications. Surgery was performed in 83 patients (53.2%), while 73 patients (46.8%) received conservative medical management. In-hospital mortality was 13.3% (11/83) among surgically treated patients and 20.5% (15/73) among medically managed patients. Although this difference was not statistically significant, the trend suggests a potential survival benefit with surgical management in selected cases.

**Figure 2:**
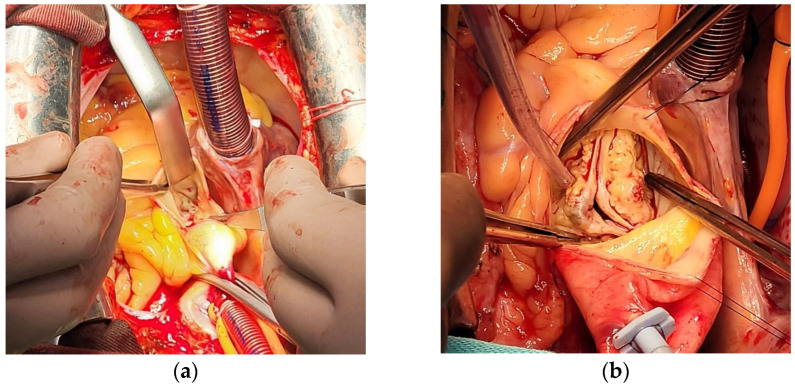
(**a**) Intraoperative aspect of infective endocarditis with marked destruction of valvular structures. (**b**) Detailed surgical exposure highlighting extensive infectious involvement and tissue damage requiring definitive surgical intervention.

Surgical treatment was typically reserved for patients presenting with severe valvular destruction, prosthetic valve involvement, heart failure, uncontrolled infection, or other major complications, in accordance with contemporary guideline-based indications for infective endocarditis. Representative intraoperative findings are illustrated in Figure 2.

**Figure 3:**
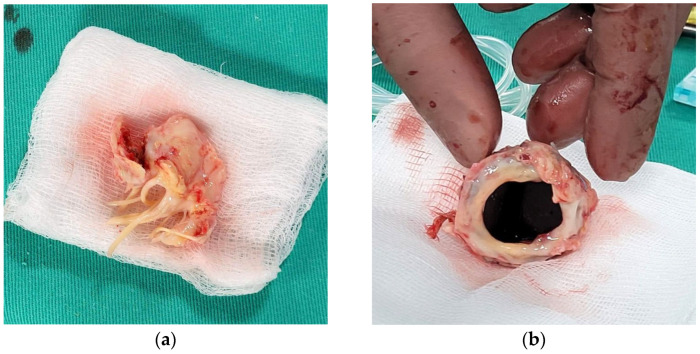
Pathological specimens obtained during surgery for infective endocarditis. (**a**) Excised native mitral valve demonstrating extensive infectious destruction with irregular, friable tissue morphology. (**b**) Removed prosthetic valvular device showing structural involvement and pathological changes consistent with prosthetic valve endocarditis.

These intraoperative findings emphasize the critical role of surgery in the management of infective endocarditis, particularly in cases characterized by extensive structural cardiac damage or life-threatening complications. However, surgical candidates were a highly selected subgroup with more advanced disease; therefore, the observed differences in mortality should be interpreted cautiously in the context of baseline severity and established surgical indications. Figure 3 presents representative gross pathological surgical specimens.

### 3.10. Clinical Outcomes and Major Complications

Major complications occurred in nearly three-quarters of patients, reflecting significant morbidity in this cohort. Cardiac complications were most common, with heart failure as the leading adverse event, often accompanied by valve-related structural damage and perivalvular extension. Extracardiac complications included acute kidney injury, systemic embolic events affecting multiple vascular territories, sepsis or septic shock, and neurological complications. These results highlight the multisystemic impact of infective endocarditis and its potential to cause severe cardiac, renal, neurological, and systemic deterioration (Table 6).

These findings reflect the complex clinical course of infective endocarditis, characterized by a high burden of both cardiac and extracardiac complications, which contribute substantially to adverse clinical outcomes.

### 3.11. Healthcare-Associated Infective Endocarditis

Healthcare-associated infective endocarditis (HAIE) was identified in 16 patients (10.3%), 136 patients (87.2%) had community-acquired IE, and 4 cases (2.6%) had unknown origin.

Patients with HAIE more frequently presented with intracardiac devices and prior invasive procedures. This subgroup showed a tendency toward more severe clinical presentation, including a higher frequency of sepsis and complications requiring surgical intervention. Even though in-hospital mortality was higher among patients with HAIE compared to those with community-acquired IE, this difference did not reach statistical significance. Although limited by subgroup size, HAIE cases appeared to present with greater clinical complexity and more frequent healthcare exposure.

### 3.12. Additional Healthcare-Associated Infections

HAIs were identified in 42 patients (26.9%) of the study cohort. Among these, 24 patients (15.4%) developed HAIs during the current hospitalization, 14 patients (9.0%) had healthcare-associated infections linked to recent prior healthcare exposure, and in 4 cases (2.6%), the source remained undetermined. A total of 48 infectious episodes were recorded, indicating that multiple infections frequently occurred in the same patient. For the distribution of HAI subtypes, percentages were calculated per infectious episode, while all other analyses were performed at the patient level.

The distribution, source, and major subtypes of HAIs are summarized in Table 7.

Gastrointestinal and respiratory infections constituted the predominant categories of healthcare-associated infections (HAIs), while device-related infections, particularly catheter-related bloodstream infections, also contributed significantly to the overall burden. The elevated frequency of HAIs linked to current hospitalization and prior healthcare exposure highlights the cumulative effects of repeated healthcare contact, extended hospitalization, invasive procedures, and broad-spectrum antimicrobial use in this population.

The microbiological spectrum of invasive HAIs included Gram-negative pathogens such as *Klebsiella pneumoniae*, *Acinetobacter baumannii complex*, and *Stenotrophomonas maltophilia*, as well as *Staphylococcus species*.

These findings further support the central role of healthcare exposure and invasive devices in the development of secondary infections.

### 3.13. Clinical Impact and Exploratory Factors Associated with Healthcare-Associated Infections

HAIs were associated with more complex clinical courses and greater healthcare resource utilization. Patients with HAIs had significantly longer hospital stays than those without HAIs (21.7 vs. 13.5 days). In-hospital mortality was also higher among patients with HAIs (21.4% vs. 14.9%; OR 1.56), though this difference was not statistically significant, possibly related to limited statistical power.

Exploratory multivariable analysis showed that central venous catheter use was associated with increased HAI occurrence (adjusted OR 3.89, 95% CI 1.08–14.06; *p* = 0.038), underscoring the importance of invasive vascular access in HAI development. Surgical treatment (adjusted OR 1.13; *p* = 0.781), age (OR 0.99; *p* = 0.505), and sex (OR 0.72; *p* = 0.442) were not significantly associated with HAI occurrence (Table 8). Due to the limited number of HAI cases and potential model overfitting, these results should be interpreted with caution and viewed as hypothesis-generating.

Exploratory subgroup analyses suggested higher mortality among patients with respiratory, urinary, and device-related HAIs. However, these findings should be interpreted with caution due to small subgroup sizes and multiple infectious episodes in some patients. Overall, HAIs were linked to longer hospitalizations and increased clinical complexity. Invasive devices, especially central venous catheters, appeared to be important factors in their development.

### 3.14. Factors Associated with In-Hospital Mortality

The overall in-hospital mortality rate was 16.7% (26/156).

In univariate analysis, mortality was significantly associated with markers of severe systemic illness, organ dysfunction, and the need for invasive supportive measures. Acute kidney injury, sepsis, leukocytosis, and healthcare-associated infective endocarditis present at admission were all significantly associated with increased in-hospital mortality. Similarly, central venous catheterization, urinary catheterization, and mechanical ventilation were more frequently observed among non-survivors, likely reflecting advanced disease severity and critical clinical status (Table 9).

Patients with embolic events (42.3% vs. 23.8%), heart failure (84.6% vs. 67.7%), and chronic kidney disease (50.0% vs. 31.5%) exhibited higher mortality rates; however, these associations were not statistically significant.

A multivariable logistic regression model was constructed to evaluate independent predictors of mortality, incorporating clinically relevant variables that were statistically significant in univariate analysis. In this model, acute kidney injury and sepsis remained independently associated with in-hospital mortality. By contrast, the associations with invasive supportive measures were attenuated, indicating that these variables primarily reflected the severity of critical illness rather than serving as independent causal predictors.

Surgical treatment was performed more frequently in survivors than in non-survivors (55.4% vs. 42.3%), suggesting a potential benefit in selected patients; however, this difference was not statistically significant.

Systemic infection severity and organ dysfunction were the primary drivers of in-hospital mortality, whereas invasive supportive therapies predominantly served as indicators of advanced clinical deterioration.

## 4. Discussion

This retrospective, single-center study of 156 patients with infective endocarditis provides a comprehensive analysis of epidemiological characteristics, microbiological findings, complications, healthcare-associated infections, and in-hospital outcomes in a contemporary tertiary cardiovascular referral cohort. The observed in-hospital mortality rate of 16.7% aligns with recent reports, which typically range from 15% to 30% [1,3,11]. This mortality rate likely reflects the impact of specialized, multidisciplinary management and a high rate of surgical intervention among selected patients, both of which are associated with improved outcomes [13,14].

The demographic and clinical profile of this cohort, characterized by predominantly male patients with a mean age of 58.5 years and a significant prevalence of cardiovascular disease, chronic kidney disease, and diabetes mellitus, is consistent with current epidemiological trends. These trends indicate a shift from younger patients with rheumatic disease to older populations with multiple comorbidities [1,3,15,16]. The predominance of degenerative valvular disease and prior IE as predisposing conditions further supports the ongoing transition from classical rheumatic etiologies to degenerative and healthcare-associated forms of IE [4].

Microbiologically, streptococci were the most frequently identified pathogens, followed by enterococci and staphylococci. This distribution differs from several recent cohorts where *Staphylococcus aureus* is predominant [6,7,8,17], potentially reflecting regional epidemiological variation, referral patterns, and a substantial proportion of patients exposed to antimicrobial therapy before admission. Pre-admission antibiotic exposure was common, often involving multiple empirical regimens initiated at referring healthcare facilities, and likely contributed to both the relatively high proportion of culture-negative IE and the frequent need for broad-spectrum empirical antimicrobial strategies.

Antimicrobial heterogeneity was primarily driven by pre-admission treatment exposure and culture-negative disease, limiting regimen-specific outcome assessment. Broad-spectrum empirical therapy was frequently required initially, with subsequent adjustments based on microbiological and susceptibility data when available. Treatment variability limited direct assessment of regimen-specific effects on outcomes, whereas pathogen virulence, host comorbidities, and complication burden appeared to exert greater influence on prognosis.

Clinical presentation was frequently subacute and nonspecific, with constitutional symptoms more common than classical febrile presentations. This finding underscores the diagnostic challenges of IE and may contribute to delayed recognition, particularly in patients with multiple comorbidities or prior antimicrobial exposure. Complications affected over 70% of patients, indicating a substantial systemic disease burden. Heart failure was the most common complication, followed by acute kidney injury and systemic embolic events, demonstrating that IE prognosis is influenced by multisystem inflammatory, renal, and embolic involvement in addition to valvular destruction. These findings are consistent with previous reports on the systemic embolic burden of IE [18].

A particularly important finding of this study is the high prevalence of healthcare-associated infections (HAIs), with more than one-quarter of patients affected. This rate is slightly higher than that reported in many contemporary cohorts and likely reflects prolonged hospitalization, repeated healthcare exposure, invasive procedures, and extensive antimicrobial use in this medically complex population. Central venous catheterization was the principal factor associated with HAI, highlighting the pathogenic role of invasive vascular devices [19,20,21]. Although HAIs were not independently associated with increased mortality in this cohort, they were linked to prolonged hospitalization and greater clinical complexity, indicating significant implications for healthcare resource utilization and patient burden. These findings support the need for careful stewardship of invasive devices and targeted infection-prevention strategies in the management of IE.

In-hospital mortality in this cohort was primarily associated with markers of systemic severity and organ dysfunction rather than isolated cardiac manifestations. Acute kidney injury and sepsis were the strongest predictors of adverse outcomes, consistent with previous evidence highlighting the prognostic significance of multiorgan dysfunction in IE [11,12]. Invasive supportive measures, such as mechanical ventilation and catheterization, were also associated with mortality in univariate analysis, likely reflecting the severity of critical illness rather than direct causality. Surgical treatment was more common among survivors and was associated with numerically lower mortality; however, this observation should be interpreted with caution due to probable selection bias, as surgical candidacy is influenced by baseline clinical status, operative feasibility, and disease stage.

These findings highlight several important clinical implications. Early identification of systemic severity markers, particularly sepsis and renal dysfunction, may enhance risk stratification and guide therapeutic prioritization. The significant burden of HAIs underscores the need to minimize unnecessary invasive procedures and reinforce infection-prevention practices. Furthermore, the complexity of microbiological diagnosis, the frequent occurrence of culture-negative disease, and high complication rates collectively support the importance of multidisciplinary endocarditis team management within specialized centers.

This study has several limitations. The retrospective design introduces potential information bias, and the single-center setting may restrict generalizability. The relatively modest sample size may reduce statistical power for subgroup and multivariable analyses, particularly regarding HAI-associated outcomes. Additionally, antimicrobial heterogeneity across referring centers may have influenced microbiological yield and treatment patterns. Despite these limitations, this study offers a detailed contemporary assessment of IE in a complex tertiary referral population, integrating epidemiology, microbiology, complications, healthcare-associated infections, and determinants of mortality, thereby contributing valuable regional and clinical insights to the evolving literature.

## 5. Conclusions

In this contemporary cohort, infective endocarditis remained a clinically complex disease characterized by frequent comorbidities, high complication rates, and significant in-hospital mortality.

The disease primarily affected middle-aged to older men with underlying cardiovascular disease, reflecting the shift toward degenerative and healthcare-associated forms of IE. Native valve involvement was most common, but prosthetic valve and device-related infections are increasingly important subsets.

Healthcare-associated infections were common and significantly affected the clinical course, leading to longer hospital stays and increased disease complexity. Use of invasive devices, especially central venous catheters, was the main modifiable risk factor, highlighting a key target for prevention.

Systemic disease severity, rather than isolated cardiac involvement, was the main driver of in-hospital mortality. Acute kidney injury and sepsis were the strongest predictors of poor outcomes, emphasizing the importance of multiorgan dysfunction in prognosis.

These findings highlight the need for early diagnosis, timely antimicrobial therapy, and careful management of invasive devices. They also support the implementation of multidisciplinary care models, such as dedicated endocarditis teams, to improve decision-making and patient outcomes [14]. Prospective multicenter studies are needed to refine prevention strategies and management in this high-risk population.

## Figures and Tables

**Table 2:** Laboratory findings at admission.

Parameter	Median (IQR)	Abnormality, *n* (%)
Hemoglobin, g/dL	9.65 (8.00–12.23)	Anemia *: 121 (77.6%)
White blood cell count, ×10^9^/L	13.2 (9.8–16.6)	Leukocytosis ^†^: 119 (76.3%)
Platelet count, ×10^9^/L	220 (158–350)	Thrombocytopenia ^‡^: 25 (16.0%); Thrombocytosis ^§^: 12 (7.7%)
C-reactive protein, mg/L	67.0 (29.5–140.5)	Elevated CRP ^¶^: 127 (81.4%)

* Anemia was defined as hemoglobin <13 g/dL in men and <12 g/dL in women. ^†^ Leukocytosis was defined as a leukocyte count >10.5 × 10^9^/L. ^‡^ Thrombocytopenia was defined as a platelet count <150 × 10^9^/L. ^§^ Thrombocytosis was defined as a platelet count >450 × 10^9^/L. ^¶^ Elevated C-reactive protein was defined as CRP >5 mg/L.

**Table 3:** Distribution of etiological agents among patients with infective endocarditis.

Etiological Agent	*n* (% of Total Cohort)	*n* (% of Microbiologically Confirmed Cases)
Streptococci	36 (23.1)	36 (34.3)
Enterococci	29 (18.6)	29 (27.6)
Staphylococci	28 (17.9)	28 (26.6)
Gram-negative bacteria	7 (4.5)	7 (6.7)
*Coxiella burnetii*	2 (1.3)	2 (1.9)
Culture-negative infective endocarditis	51 (32.7)	-

**Table 4:** Most frequently used antimicrobial regimens in the overall cohort.

Antimicrobial Regimen *	*n* (%)
Ceftriaxone + vancomycin	28 (17.9)
Ampicillin + gentamicin	20 (12.8)
Vancomycin + gentamicin	12 (7.7)
Ceftriaxone + gentamicin	12 (7.7)
Ampicillin + ceftriaxone	4 (2.6)
Ampicillin + vancomycin	4 (2.6)
Ampicillin + gentamicin + vancomycin	4 (2.6)
Oxacillin + gentamicin	3 (1.9)

* Only the most frequently used regimens are shown.

**Table 5:** Most frequently used empirical regimens in culture-negative infective endocarditis.

Antimicrobial Regimen	*n* (% of Culture-Negative IE)
Ceftriaxone + vancomycin	13 (25.5)
Vancomycin + gentamicin	4 (7.8)
Ampicillin + gentamicin	4 (7.8)

**Table 6:** Major complications of infective endocarditis.

Variable	Value
Any complication, *n* (%)	116 (74.4)
Heart failure, *n* (%)	110 (70.5)
Valve-related complications, *n* (%)	81 (51.9)
Perivalvular abscess/pseudoaneurysm, *n* (%)	22 (14.1)
Acute kidney injury, *n* (%)	58 (37.2)
Embolic events, *n* (%)	51 (32.7)
Splenic infarction, *n* (%)	39 (25.0)
Neurological complications *, *n* (%)	26 (16.7)
Ophthalmologic complications ^†^, *n* (%)	13 (8.3)
Sepsis or septic shock, *n* (%)	17 (10.9)
Cardiogenic shock, *n* (%)	10 (6.4)
Multiple organ dysfunction syndrome, *n* (%)	10 (6.4)

* Includes ischemic stroke and other central nervous system complications. ^†^ Primarily represented by optic neuritis.

**Table 7:** Additional healthcare-associated infections in the study group.

Variable	Value
Patients with ≥1 HAI, *n* (%)	42 (26.9)
Total HAI episodes, *n*	48
HAIs acquired during current hospitalization, *n* (%)	24 (15.4)
HAIs associated with recent prior healthcare exposure, *n* (%)	14 (9.0)
Undetermined source, *n* (%)	4 (2.6)
*Clostridioides difficile* infection, *n* (% of HAI episodes) *	17 (35.4)
Ventilator-associated pneumonia, *n* (% of HAI episodes) *	10 (20.8)
Catheter-related bloodstream infection, *n* (% of HAI episodes) *	9 (18.8)
Urinary tract infection, *n* (% of HAI episodes) *	5 (10.4)
Skin and soft tissue infection, *n* (% of HAI episodes) *	4 (8.3)
Other respiratory infections (including SARS-CoV-2), *n* (% of HAI episodes) *	3 (6.3)

* Percentages for HAI subtype distribution were calculated per infectious episode (*n* = 48), whereas all other percentages were calculated at the patient level (*n* = 156).

**Table 8:** Length of stay and in-hospital mortality in patients with and without HAIs.

Variable	HAIs	No HAIs	*p*-Value
Patients, *n*	42	114	—
Length of stay (days)	21.7	13.5	<0.001
In-hospital mortality within group, *n* (%)	9/42 (21.4)	17/114 (14.9)	0.340

**Table 9:** Univariate analysis of factors associated with in-hospital mortality.

Variable	OR	*p*-Value
Acute kidney injury	4.10	0.002
Central venous catheterization	10.71	0.003
Urinary catheterization	11.52	0.003
Mechanical ventilation	5.28	0.004
Sepsis	4.14	0.005
Healthcare-associated infection (non-IE) present at admission *	4.88	0.001
Leukocytosis ^†^	3.23	0.017

* Healthcare-associated infections present at admission referred to infections other than the index infective endocarditis episode, identified at hospital admission, and classified according to healthcare-associated infection criteria. ^†^ Defined as leukocyte count >10.5 × 10^9^/L.

## Data Availability

All data generated or analyzed during this study are included in this published article.

## Abbreviations

The following abbreviations are used in this manuscript:

AKI	acute kidney injury
CI	confidence interval
HAI	healthcare-associated infection
HAIE	healthcare-associated infective endocarditis
IE	infective endocarditis
IQR	interquartile range
MODS	multiple organ dysfunction syndrome
MR-CoNS	methicillin-resistant coagulase-negative staphylococci
MRSA	methicillin-resistant *Staphylococcus aureus*
MSSA	methicillin-sensitive *Staphylococcus aureus*
OR	odds ratio
NVE	native valve endocarditis
PVE	prosthetic valve endocarditis
